# Next Generation Air Quality Platform: Openness and Interoperability for the Internet of Things

**DOI:** 10.3390/s16030403

**Published:** 2016-03-18

**Authors:** Alexander Kotsev, Sven Schade, Massimo Craglia, Michel Gerboles, Laurent Spinelle, Marco Signorini

**Affiliations:** 1 Digital Earth and Reference Data Unit, Institute for Environment and Sustainability, Joint Research Centre, European Commission, Ispra 21027, Italy; sven.schade@jrc.ec.europa.eu (S.S.); massimo.craglia@jrc.ec.europa.eu (M.C.); 2 Air and Climate Unit, Institute for Environment and Sustainability, Joint Research Centre, European Commission, Ispra 21027, Italy; michel.gerboles@jrc.ec.europa.eu (M.G.); laurent.spinelle@jrc.ec.europa.eu (L.S.); 3 Liberaintentio Srl, Malnate 21046, Italy; marco.signorini@liberaintentio.com

**Keywords:** interoperable sensors, INSPIRE, AirSensEUR, Internet of Things, sensor observation service, sensor web enablement, air quality

## Abstract

The widespread diffusion of sensors, mobile devices, social media and open data are reconfiguring the way data underpinning policy and science are being produced and consumed. This in turn is creating both opportunities and challenges for policy-making and science. There can be major benefits from the deployment of the IoT in smart cities and environmental monitoring, but to realize such benefits, and reduce potential risks, there is an urgent need to address current limitations, including the interoperability of sensors, data quality, security of access and new methods for spatio-temporal analysis. Within this context, the manuscript provides an overview of the AirSensEUR project, which establishes an affordable open software/hardware multi-sensor platform, which is nonetheless able to monitor air pollution at low concentration levels. AirSensEUR is described from the perspective of interoperable data management with emphasis on possible use case scenarios, where reliable and timely air quality data would be essential.

## 1. Introduction

The ways in which we create, manage and make use of data is fundamentally changing under the influence of several interdependent factors. For Earth sciences, this is similar to the revolution caused by the use of remote sensing data during the 1970s [1]. The number of devices, interconnected into the Internet of Things (IoT) is expected to reach 50 billion in 2020 [2]. Volunteers, also referred to as citizen scientists [3], empowered by inexpensive and readily available technology, are increasingly engaged in collecting and processing heterogeneous data, which has traditionally been collected by authoritative sources. In particular, in the field of air quality, many recent citizen science initiatives, such as [4,5,6], aim to complement and/or substitute official measurement networks in their attempt to monitor the quality of ambient air. The approaches that those projects adopt are different, but they all rely on inexpensive hardware and establish a community of volunteers who are engaged in collecting observation data. While those activities achieve very good results in raising visibility and engaging citizens on the importance of air quality, they are still not able to provide sufficient quality for observation data.

That is why we consider that observation data collected by citizens, without a means to estimate the quality of observation data and/or compare to existing authoritative sources of information, should not be used as input to modeling and/or for decision making. At the same time, stations belonging to existing authoritative air quality networks are not dense; equipment is expensive; and the majority of available information technology solutions for data collection and exchange is vendor specific. It is thus difficult to combine observation data from different channels. Furthermore, interpolation techniques used in air quality modeling are usually country dependent, and not open enough, thus acting as “black boxes” with results that are very difficult, if at all possible, to evaluate and reproduce. For this reason, being able to provide good quality data with high granularity (e.g., at the street level) and mashing-up air quality data from heterogeneous sources is still challenging, particularly in urban and suburban areas [7].

Within this context, the Joint Research Centre of the European Commission (JRC) is working on the AirSensEUR project, which aims at the establishment of an affordable (under 1000 euro) open software/hardware multi-sensor platform, which is nonetheless able to monitor air pollution at low concentration levels. This manuscript describes the AirSensEUR platform from the perspective of spatial data infrastructures (SDI) and interoperable data management. We do not attempt to provide an exhaustive overview of the air quality-specific hardware configuration, as this is already done by Gerboles *et al.* [8,9].

The second section of the manuscript defines the theoretical foundation for the implementation of the platform, with particular emphasis on the research challenges for the establishment of open and transparent sensor networks interconnected through the means of the IoT. The third section provides an overview of the interoperable components of AirSensEUR, which we have intertwined to provide a single “plug-and-play” bundle capable of producing reliable observation data in different contexts. In Section 4, we describe several application scenarios for AirSensEUR, in particular for: (i) regulatory; and (ii) informative purposes. Finally, we conclude with the lessons learned, remaining challenges and the direction of our future work.

## 2. Context

**Figure 1:**
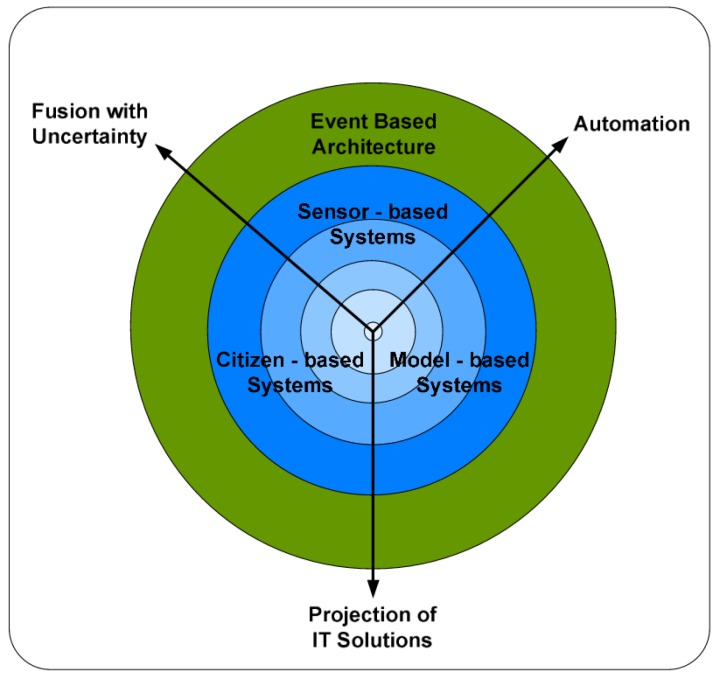
Theoretical challenges in sensor web research [11].

Pervasive computing and citizen science provide completely new channels for environmental sensing. Official data can be complemented and even substituted through citizen-driven initiatives. This process is however not straightforward. The integration and fusion of data from different sources, which were acquired in different contexts with heterogeneous methods and tools, face serious interoperability issues on the technical, semantic, organization and legal levels [10]. Schade and Craglia [11] outline several challenges bounding the future development of the sensor web (Figure 1). Whereas the concentric blue circles in the center of the figure illustrate the need to address different data aggregation levels (beginning with raw data in the very middle), an event-based architecture will be required to unite all datasets and streams, independent of their origin, this being sensor measurements, modeling results or people’s observations. Three transversal challenges are cross-cutting through the figure: (i) the automation (optimized machine support) of underlying processes; (ii) the projection/re-application of general IT solutions (e.g., to address security and privacy issues); and (iii) data fusion/integration, including the propagation of uncertainties throughout the applied algorithms.

Schade and Craglia provide a theoretical framework to address some of the key issues arising in the establishment of sensor networks. The authors apply the notion of the sensor web as an integrating concept addressing the common consideration of measurements and observations, independent of their distinct origin. In this way, outputs from authoritative networks (e.g., those of environmental protection agencies), scientific prediction and forecasting models (e.g., for the dispersion of air pollution, emission of pollutants into natural resources or the effects of climate change) and from citizens (see also the citizen-based systems section, below) can be seamlessly integrated. Although these multivariate sources can be integrated conceptually and examples of this application exist [12,13], a series of practical challenges still remain. A list is provided in Section 2.1.

We consider that our work on AirSensEUR addresses together the majority of those challenges; thus, the lessons learned, and our proposed approach, if adopted, would lead to more open, transparent and interoperable sensor network infrastructures.

### 2.1. Citizen-Based Systems

Many citizen science projects, such as [4,5,6], take advantage of the rapidly developing field of mobile low cost sensors. They address data-related issues from different perspectives (e.g., smart cities, Internet of Things, digital single market, citizen science), and at different levels (local, national, international).

There is also an emerging movement of projects initiated and developed by individuals or groups that do not have any affiliation with the scientific establishment. This do-it-yourself (DIY) movement has been paving the way for the next steps for citizen science. Anyone who is fascinated or curious about science now finds a lower threshold to enter expert realms, facing DIY options, tools and spaces to build anything from scientific instruments for environmental measurements and for genome sequencing to satellites and other machines or devices. Low cost sensors (for instance, CO${}_{2}$, light intensity, sound or humidity), several programming languages, open-source hardware prototyping platforms or microcontrollers (such as Arduino or Raspberry Pi) have become adaptable, modular and easy to use at the starter level. A wider ground for experimentation emerges when these solutions are coupled with access to digital tools (especially 3D printers) and hands-on activities in shared spaces. In addition, connection with on-line communities and access to web-based tutorials and documentation in repositories, such as Instructables or GitHub, facilitate the establishment of networks of support and collaboration with others with common interests and increase science literacy.

Notwithstanding these positive developments, the use of low cost sensors by citizens still faces major challenges, which limit the establishment of scientifically-sound results. Those are described in [7] and include:

- Difficult discovery of environmental sensor devices and networks, due to the lack of metadata and services that expose them;
- Spatial/temporal mismatch of observations and measurements deriving data from unevenly-distributed monitoring stations that do not always form networks causing difficulties in data reuse for initially-unintended purposes;
- Lack of interoperability between components (e.g., measurement devices, protocols for data collection and services) of acquisition and dissemination systems;
- Information silos, created by the use of standalone vocabularies that are bound to particular environmental domains, such as hydrology and air quality;
- Proprietary solutions for logging sensor measurements, which require custom code to be wrapped around the manufacturer’s software development kit;
- Accuracy of the pollution sensors, which, as described in [14], is the major fault in any environmental network of sensors due to their low sensitivity to ambient levels of air pollutants.

### 2.2. International Standards

To address these issues, we present in Section 3 the AirSensEUR open source platform. Its development leverages the increased convergence of international standards in the geographic and telecommunication domains (IEEE, Open Geospatial Consortium—OGC, International Telecomunication Union—ITU) [15] and the development of the European Spatial Data infrastructure (INSPIRE). The latter [16] is unlocking heterogeneous data, produced by public sector organizations in 28 European countries. Relevant work on sensors in INSPIRE covers both data encoding [17] and network services [18], together providing all necessary means for “plugging” spatio-temporal data into SDIs, thus enabling its use and reuse combined with other relevant resources [7].

## 3. AirSensEUR: An Interoperable Plug-and-Play Sensor Node

In order to advance this research on citizen-based observation systems, and using the latest standards available, we developed an interoperable plug-and-play sensor node: AirSensEUR. It is designed as an open platform based on several pillars, which ensure that individual sensor nodes are capable of interoperating with heterogeneous sources of data. The high level objective, which determines the bounding conditions of AirSensEUR, is to design and build a platform that: (i) is capable under certain conditions of producing indicative observation data that meet the legal requirements of the EU Air Quality Directive [19]; and (ii) implements a download service, as required by the EU INSPIRE Directive [16].

**Figure 2:**
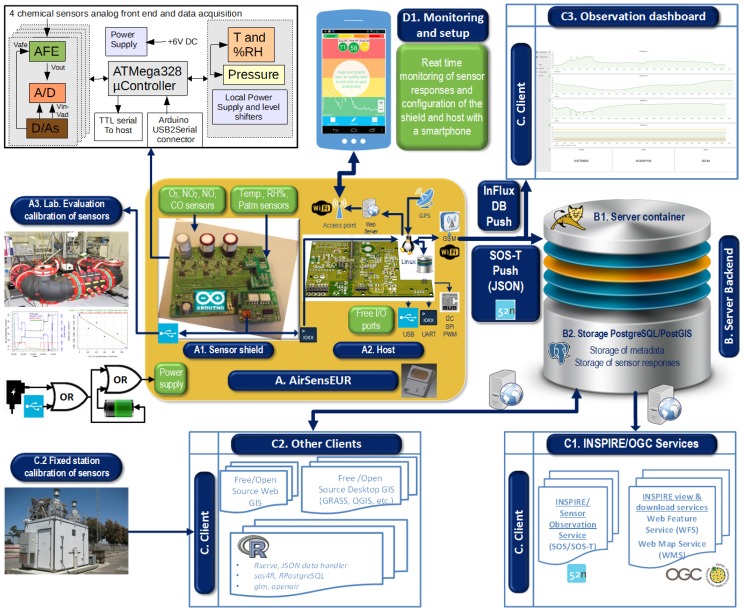
Architecture of AirSensEUR.

The platform itself consists of a bundle of software and hardware (Figure 2), which are configured to work together in a synchronized manner. The hardware (Subsystem A) consists of a sensor shield and host, further described in Section 3.1, while the software components being used are described in Section 3.2, both in terms of backend (Subsystem B) and client applications (Subsystem C). Further information about the platform is available online at [20].

### 3.1. Open Hardware

**Figure 3:**
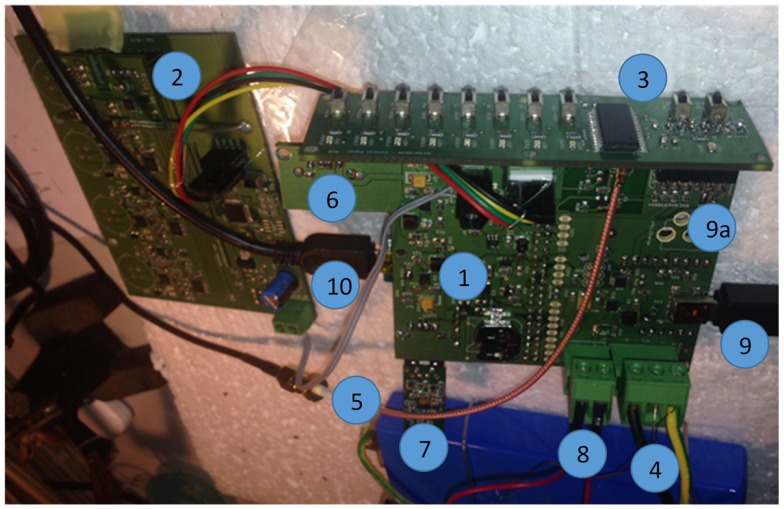
Hardware components of AirSensEUR.

In terms of hardware, the platform (Figure 2) consists of a multi-sensor shield (A1), which is connected to a Linux-based host (A2). The individual components of AirSensEUR are shown in Figure 3 and described in further detail within Table 1. AirSensEUR documentation, together with computer-aided designs of boxing for 3D printing, is open by design, thus ensuring the ability to reproduce and reuse the results. All resources are made available at [20,21].

Currently, one shield with four amperometric sensors and an ancillary board with temperature, humidity and pressure sensors have been developed for AirSensEUR. The long-term objective is to interest the scientific community in validating and further developing additional shields for other pollutants (e.g., measuring particulate matter (PM)). Shields might be connected through one of several available communication (COM) ports of the platform.

The AirSensEUR shield is a high precision four-channel three-electrode sensor board. It also includes a daughter board with temperature/humidity (UR100CD, Technosens-IT) and pressure (BMP180, Bosch-DE) sensors together with I2C level shifters to interface to the ATMega328 microcontroller managing the shield. Each sensor channel is composed of a fully-programmable analog front end (AFE, TI LMP91000, Texas Instruments, U.S.), a 16-bit analogue to digital (A/D) converter (TI ADC16S626) and a 12-bit digital to analogue (D/A) converter (AD5694RB). The D/A converter dynamically sets the range of the A/D converter in order to keep the converter resolution in the sensor output range, making AirSensEUR suitable to measure extreme low voltages (15-μV resolution on a range set to ±0.5 V), as needed with the sensitivity of the selected sensors. The ATMega328 controls the AFE of the sensor channels, A/D and D/A registers, daughter board for ancillary data. It then retrieves, filters and averages the responses of the seven sensors and concatenates all into a hexadecimal string. The ATMega328 receives a firmware developed in the Arduino framework and Integrated Development Environment (IDE) through a serial line on the shield.

A USB board accommodated on the shield allows real-time data acquisition of AirSensEUR data for laboratory calibration. Additionally, a communication protocol and a Java control panel have been developed in order to easily configure the AFE of each channel (sensor voltage, D/A outputs in order to fix A/D conversion limits, gain of the signal, load resistance of each sensor (RL), bias, Infinite Impulse Response (IIR) filtering [22], data acquisition periodicity and averaging time) and read sensor responses. To the best knowledge of the authors, the AirSensEUR shield is among the boards with the widest control by the user of all sensor parameters, allowing maximum flexibility. The schematic representation of the chemical sensor board is given on the upper left corner of Figure 2.

So far, tests have been conducted with four City Technology Sensoric sensors: O${}_{3}$ 3E1F, NO${}_{2}$ 3E50, NO 3E100 and CO 3E300 [23]. However, the shield can accommodate other two and three-electrode amperometric sensor brands and models, including:

- the Sensoric model (diameter of 16 mm, mounted with a TO5 connector),
- sensors with a 20-mm diameter: the 4 series of City Technology or SGX Sensortech [24], the “miniature” series of Membrapor [25] and the A sensor series of Alphasense [26]),
- and sensors with a 32-mm diameter: e.g., the 7 series of City Technology or SGX Sensortech, the Membrapor “Compact” sensor series or the “B” sensor series of Alphasense.

The sensor host (A2 in Figure 2) is based on the Arietta G25 (ACMESystem-IT) and consists of a low cost Linux embedded module CPU Atmel (400 MHz ARM9^TM^ processor) loaded with 256 MB of DDR RAM. It also accommodates other devices: a 32-GB SD card with pre-installed Linux, a GPS, a GPRS and a WiFi access point.

Power supply comes from either a battery or through USB/power line. The power budget of AirSensEUR was estimated summing power requirements for each individual subsystem. For the shield with four sensors and the ancillary daughter board, 20 mA@5 V was measured; 70 mA@5 V is required by the ARM module of the Arietta, 20 mA by the GPS and 15 mA by the optional external active antenna. This aggregates to a steady total of 125 mA@5 V (0.625 VA). Introducing possible losses generated by switching power supplies, with efficiencies up to 80%, we expect a consumption of 0.780 VA. A 20-Ah, 3.3-V (64 Wh) single cell Lithium iron phosphate battery (LiFePO4) will be able to power up the system for more than 80 h. Measurements done when sending data through the GPRS dongle would however yield an average consumption of 300 mA@5 V (1.5 VA). With an estimated session time of 30 min and introducing losses caused by the switching power supply, this generates an estimated 1 Wh for each data session. Planning four updates a day requires 4/5 Wh, thus reducing the expected overall running time to about 60 h depending on external conditions, mainly due to (i) temperature and (ii) battery life.

### 3.2. Open Source Software

We used open source software in order to take advantage of the rapid development cycle and outreach to existing communities. The server side component of the platform is by design based on OSGEO-Live—the free and open source bundle of the Open Source Geospatial Foundation [27]. This provides many opportunities, as data can be further used within both web and desktop Geographic Information System (GIS) clients. Furthermore, through using OSGEO-Live as the software environment for handling data from AirSensEUR, we ensure that the open source projects that we use are supported by a healthy community and meet baseline quality criteria [28]. The components that are chained together in AirSensEUR are provided in Table 2.

The orchestration of individual open source tools is described in the subsections below. For clarity, the overview is split into: (i) sensor host; (ii) server components; and (iii) clients.

#### 3.2.1. Sensor Host

A set of Java programs retrieves data from the shield and the GPS. Together with the timestamp, these data are added to a local sqlite3 database (A2 in Figure 2), stored on the SD card of the Arietta. Finally, the data of the local database are pushed via GSM/GPRS to an external server through standardized JSON requests, based on a transactional sensor observation service (SOS-T). This functionality for web transactions is provided by the JSON binding of the $52{\;}^{\circ }\text{North}$ SOS implementation, described by [30].

**Figure 4:**
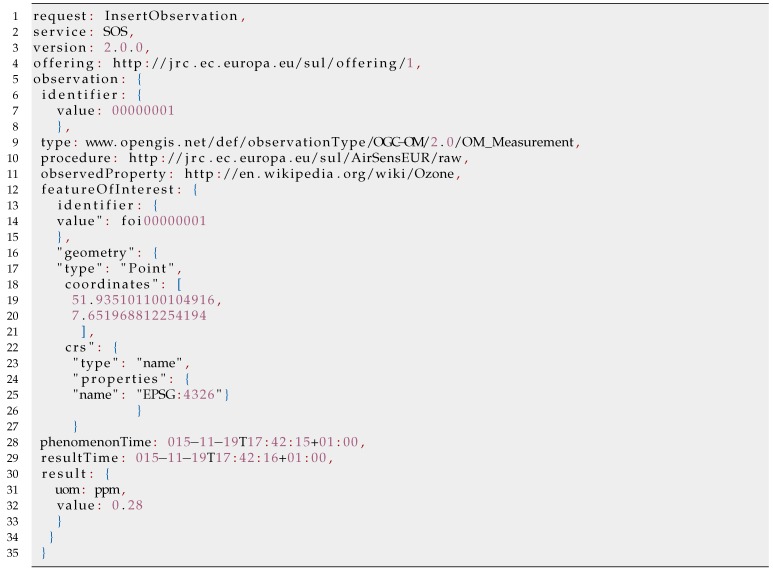
Sample *InsertObservation* JSON request.

The use of SOS-T as the means for the migration of data from the sensor host to the server provides us with several significant advantages over a direct web access to the AirSensEUR database. Those include (i) high level of security (the sensor host does not provide credentials for access to the database, and *InsertObservation* requests are limited to a predefined number of IP addresses), as well as (ii) independence from the database schema. Furthermore, the JSON syntax of the request is minimalistic in terms of size and is therefore well suited for the transmission of big volumes of observation data. A sample *InsertObservation* request is provided in Figure 4.

#### 3.2.2. Server Components

An SOS exposes observation data in an interoperable manner, so that it can be retrieved and directly re-used by standard clients without any need to adopt an access protocol or data structures on the consumer side. Such functionality is, for example, fundamental in order to integrate citizens’ observations with institutional measures on-the-fly. Through SOS, the platform implements by design an INSPIRE download service and “plugs” data into spatial data infrastructures (SDI), established as a result of the implementation of the European INSPIRE Directive [16]. This is possible because the SOS implementation that is used within the platform is already extended as an INSPIRE download service [18]. This provides numerous opportunities for combined use of data, e.g., for analysis of air quality together with the distribution of population or species, thus trying to understand the effect of pollution on human well-being or species.

#### 3.2.3. Clients

**Figure 5:**
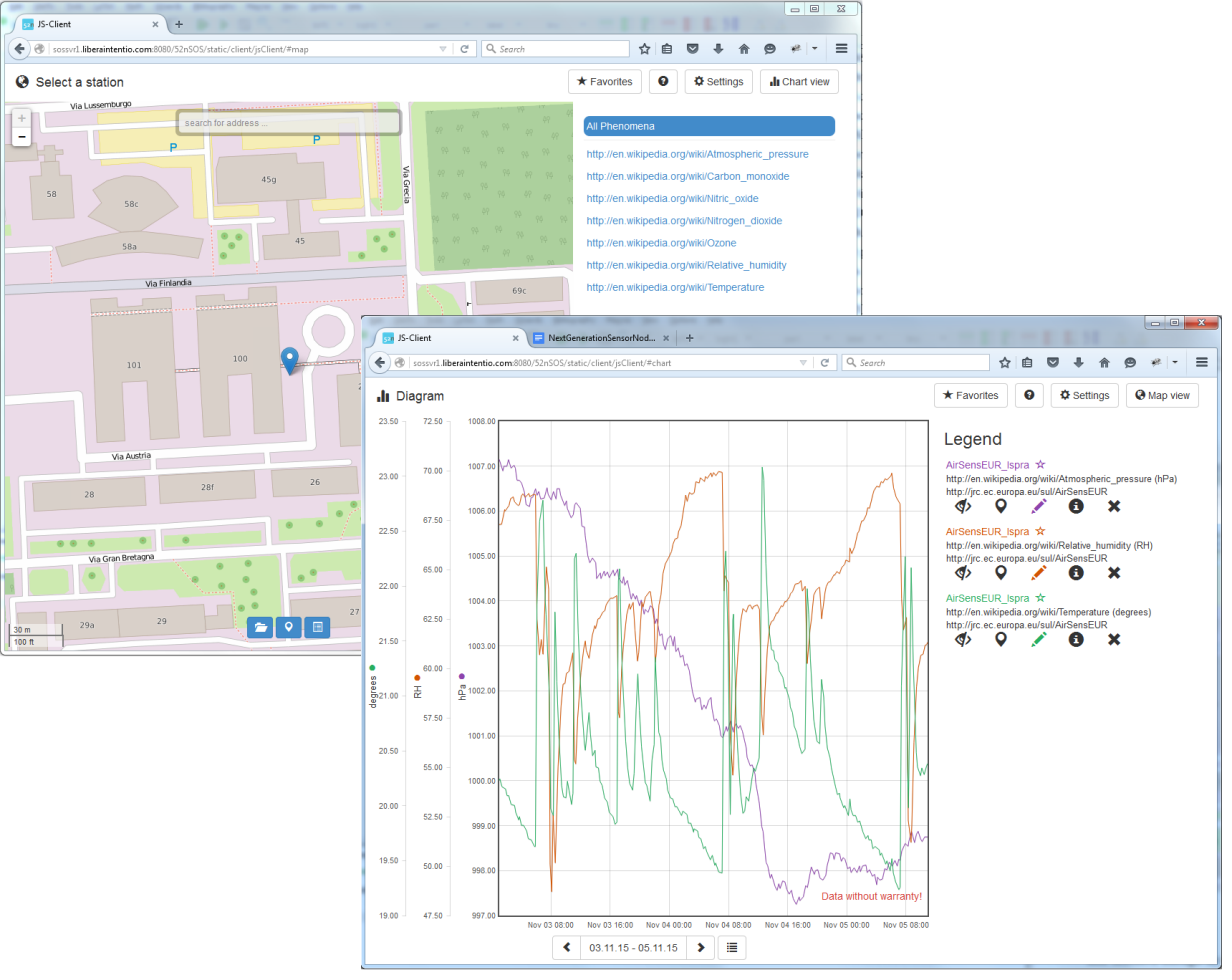
User interface of the $52{\;}^{\circ }\text{North}$ SensorWeb client, loaded with AirSensEUR data.

The SOS-based web service allows direct interaction with the observation data through standard (POST, GET) requests. That is why the only precondition for interaction with the AirSensEUR server is a web browser and some basic knowledge of the SOS interface standard. Observations in SOS can also be consumed by an increasing number of desktop (e.g., QuantumGIS) and web (e.g., OpenLayers, $52{\;}^{\circ }\text{North}$ SensorWeb client, ESRI ArcGIS for Server, RStudio server) clients, which makes the retrieval of data even easier. The $52{\;}^{\circ }\text{North}$ SensorWeb client (Figure 5) is the main means for communication of observation data from AirSensEUR, as it provides an easy to use environment, which is also mobile friendly.

**Figure 6:**
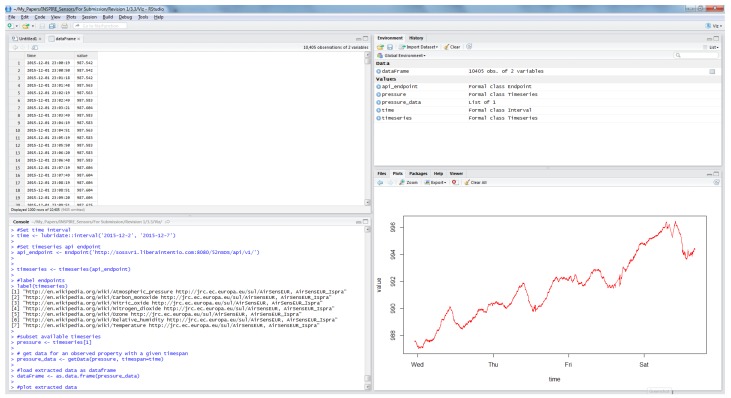
AirSensEUR data loaded and visualized in “R”.

Furthermore, data from AirSensEUR can be pulled directly from the console environment of the “R” statistical package (Figure 6) through the sensorweb4R library [31]. This provides numerous opportunities for additional processing (e.g., calibration) and visualization.

## 4. Use Cases

The AirSensEUR platform, as just detailed above, is designed to enable a rich portfolio of possible applications. In the section, we illustrate the potentials of our solution by providing application examples where reliable and timely air quality data are absolutely essential. Within this context, we distinguish two types of applications, related to (i) the monitoring of air pollution for regulatory purposes; and (ii) other applications for informative purposes.

### 4.1. Monitoring for Regulatory Purposes

In Europe, the mandatory monitoring of air pollution is managed by the European Directive for Air Quality [19]. This Directive, which does not consider mobile monitoring, but only fixed measurements, sets different categories of measurement methods according to the data quality objectives (DQOs) they can meet. The DQOs set maximum levels of measurement uncertainty that each method shall meet at limit values, defined for each pollutant based on health effects. The Directive establishes a framework of methods for air pollution monitoring for regulatory purposes as presented here:

- reference methods that can be applied everywhere and for all purposes with a maximum measurement uncertainty of 15% for O${}_{3}$, NO${}_{2}$, NO${}_{x}$ and CO;
- indicative methods that can be applied in areas where a defined level, the upper assessment threshold (UAT), is not exceeded, and they permit a reduction of 50% of the minimum reference measurements where the UAT is exceeded, thus allowing one to diminish the cost of monitoring by reducing the mandatory number of reference methods. Indicative methods are associated with a DQO of 25% for NO${}_{2}$, NO${}_{x}$ and CO, 30% for O${}_{3}$;
- objective estimation that can only be implemented in an area of low levels of air pollution with a DQO of 75% for O${}_{3}$, NO${}_{2}$, NO${}_{x}$ and CO.

Recently, several evaluations of sensor performance were performed, including both laboratory and field experiments [32,33,34,35]. With these results, low cost sensors are not able to meet the DQOs of hourly reference measurements set in the Air Quality Directive. Conversely, these evaluations suggest that some sensors could reach the DQOs for indicative measurements. We expect that AirSensEUR can meet the DQOs of indicative measurements for some compounds. These DQOs are about half less stringent than the one of reference measurements for O${}_{3}$, NO${}_{2}$, CO and SO${}_{2}$. The first protocol of evaluation of sensors for indicative measurements has been developed [36]. It is currently used by the European Committee for Standardization—CEN (Technical Committee 264 on air quality—Working Group 42 on sensors), which is currently drafting such a protocol.

### 4.2. Monitoring for Informative Purposes

#### 4.2.1. Fixed Measurements

Views are currently evolving the thinking that the presented legislative framework is not completely fit for the use of low cost sensors. In particular, a new method category “informative methods” not linked with DQOs would be beneficial in order to allow for simpler and faster evaluations. The aim would be to base these evaluations only on field tests by comparing co-located sensors with reference methods. Recently, the South Coast Air Quality Management District of California (USA) released a number of these comparisons [37] using the coefficient of determination (R^2^) as the main indicator of the quality of sensor values. Spinelle *et. al.* [38] proposed to use a target diagram [39] to easily compare the performances of sensor measurements.

That is why the usefulness of fixed informative measurements with lower DQO, prescribed by the European Directive, remains an open question. Nevertheless, low cost sensors carry a number of advantages compared to reference measurements. Sensors, including AirSensEUR, are less expensive than reference methods, allowing them to be deployed in dense networks and to provide detailed information with larger spatial coverage than the one of traditional monitoring stations. For example, within the RESCATAME (Pervasive Air-quality Sensors Network for an Environmental Friendly Urban Traffic Management) project [40], sensors were installed at 35 points on two busy streets of Salamanca (Spain), and each point was equipped with seven sensors: CO, NOx, O${}_{3}$, fine particles (PM), noise, humidity and temperature. The sensors were used to simultaneously assess air pollution and to monitor traffic. Based on this information, prediction models estimated the level that air pollution could reach in the next one and three hours. This allowed the traffic department to foresee high pollution episodes and act accordingly. High air pollution estimates triggered changes in the timing of traffic lights, temporary blocking of a lane or regulations imposed by local police officers. Other projects with fixed low cost air quality sensors aim at increasing the spatial and temporal scale of information in highly granular environments, *i.e.*, in areas that are spatially heterogeneous with variable emission sources. For example, within the SNAQ (Sensor Networks for Air Quality Heathrow) project [41], a network of 50 sensors was installed around Heathrow airport. Emissions inventories and dispersion model results were improved using the sensor data. In addition, source apportionment was studied around the airport through the use of sensor data.

Other applications of sensors at fixed sites include monitoring in remote areas where power supply is not readily available because of their limited needs in electricity and the absence of required routine maintenance. The assessment of concentration gradients or alerts and industrial fence line monitoring within industrial areas where high pollution levels are expected has been a typical area of application for low cost sensors for decades.

#### 4.2.2. Mobile Measurements, Outdoor/Indoor Environments and Citizen Observatories

Exposure to air pollution and the associated health risks are tightly related to the spatial and temporal occurrence of individual activities. There is an increasing body of knowledge that evaluates the human exposure to air pollution [42]. Significant variations are identified in the exposure, even between individuals from the same household [43]. Still, the integration of the spatio-temporal dynamics of pollution together with the spatio-temporal trajectories of individuals into a suitable analytical framework is challenging [44].

Within this context, a major advantage of low cost sensors, such as AirSensEUR, is their portability, which together with their limited needs of power supply allows a number of mobile applications generally aimed at monitoring direct population exposure to air pollution. This is a unique feature of sensors that is generally not possible to achieve with reference methods.

The EU FP7 project Citi-Sense is developing a sensor-based Citizens’ Observatory Community to improve the quality of life in cities [45]. In this project, citizens are proposed to contribute to and participate in environmental governance by using novel technologies as sensors. A number of Citizen’s Observatory Projects of this type have been implemented in which mobile monitoring is carried out by citizens, for example Common Sense, the forerunner of this type of project [46,47], and Citi-Sense [48,49].

An exhaustive review of these types of projects can be found in [50]. It is worth mentioning Citi-Sense-Mob [51], which aims at using sensors mounted on buses and bikes combined with models and monitoring stations to produce personalized data as alerts and exposure through web and smartphones applications. The OpenSense project [52] also used mobile monitoring on buses to produce high spatially-resolved maps of pollution distribution. In this type of project, mobile monitoring is restricted to outdoor measurements. This is an important aspect for the data quality of measurements with sensors. It is more difficult to control the data quality of measurements that are carried out moving very fast from outdoor to indoor environments, which is typical when sensors are worn. In fact, sensors are generally strongly affected by the rapid change of air composition, temperature and humidity, which are typical for mobile applications going from outside to indoors. That is why the speed effect, associated with the movement of sensors, must be considered throughout the whole life-cycle (design, deployment and analysis) of a measurement campaign [53].

AirSensEUR has been designed so that it can be used in all of the applications presented above. The interoperability of data and the power supply by both a 220-V socket and long-autonomy battery allow for fixed and mobile measurements for regulatory, informative or citizen observatory projects. Moreover, a specialized algorithm using NMEA (National Marine Electronics Association) traces to determine outdoor and indoor environments is also in development. It could be used for: (i) health-related information on population exposure; and (ii) for the application of new ranges of calibration functions.

### 4.3. Strategies to Ensure Data Quality of AirSensEUR

The major limitation of the diffusion of low cost sensors in the last few years has been the questionable quality of observation data. Once the design of the AirSensEUR prototype has reached a satisfactory state, we will be working on a procedure for calibration. The list of parameters that affect the electrochemical sensor responses is now well known and includes: cross sensitivities to gaseous interfering compounds, long-term drift, temperature and humidity effects. We have already foreseen the different possible routes towards an effective procedure for calibration:

- establishing a deterministic model based on laboratory and field experiments based on a strict protocol of the sensor test [32];
- as the AirSensEUR includes seven sensors, cross sensitivities may be solved in a multivariate system of equations;
- design of an active sampling system on top of the sensors to easily control the humidity of the air beam and to filter the gaseous interfering compounds;
- calibration at the field monitoring station using co-located pair of reference and sensor data. The types of calibration methods can include linear, multi-linear equations, sensor cluster coupled with artificial neural network (ANN), *etc*. A good comparison of these techniques is given in [38]. ANN was found to be the most effective technique though requiring additional metal oxide (MOx) sensors not yet present on the AirSensEUR shield;
- in the case of mobile sampling, a few algorithms have been developed for the re-calibration of mobile sensors *versus* reference measurements or recalibration of sensors *versus* freshly-calibrated sensors in a mobile environment [52,54];
- future development of calibration facilities (including zero and span) directly on the sensor platform can be imagined. This solution, likely expensive, may only be adopted in association with the active sampling system a few points above. Both of them would use the same pneumatic system. While designing zero air using selective chemical filters seems possible, for example thriethanolamine (TEA) for NO${}_{2}$, 1,2-di(4-pyridil)-ethylene (DPE) or indigo for O${}_{3}$, the development of a span gas generator appears quite challenging.

## 5. Discussion and Conclusions

Following initial testing of the AirSensEUR platform of approximately 2.5 months, including the collection from one shield of 4.5 million observations (seven observed properties, collected every 10 s), we consider that AirSensEUR is easy to configure and expect it to be sensitive enough to measure ambient air pollution in the range expected at background and traffic sites placed in rural, urban and suburban areas. The authors would like to point out that the manuscript presents a platform, rather than the performances of sensors. In effect, the list of sensors that can be mounted on AirSensEUR (approximately 230 sensors, as described in [9]) is too long to be tested. An example of an application with the CityTech sensors, O${}_{3}$ 3E1F, NO${}_{2}$ 3E50, NO 3E100 and CO 3E300 is given in [8,9]. The references show by calculation and experiments that the combination of sensors with the AirSensEUR platform allows one to reach an electronic resolution of 15.3 μV for individual measurements [9]. This resolution corresponds to detection limits of respectively 3.2 ppb·min, 16.7 ppb·min, 74.9 ppb·min and 0.056 ppm·min for the cited CityTech sensors. Lower limits of detection would be obtained with sensors that are more sensitive. The sensitivity of CityTech sensors allows monitoring O${}_{3}$, NO${}_{2}$ and CO when averaged over one hour as required by the European Directive for Air Quality [19].

The platform provides a promising technological approach for the monitoring of population exposure in mobile context. What makes AirSensEUR different from other similar solutions is:

- “Plug-and-play” architecture, which is transparent, allows configuration of each individual component and can be adapted to different mobile and *in situ* use cases;
- First low cost sensor, expected to provide indicative measures, which would be within the bounds of the requirements of European Union’s Air Quality Directive [19];
- Interoperable architecture, which is aligned by design with the legal requirements of the European Union INSPIRE Directive [16], thus being able to “plug” data into the rapidly evolving pan-European spatial data infrastructure;
- Possibility for the establishment of an open software/hardware/data community around the project through the adoption of a transparent approach, combined with the broad use of well-established open source technology;
- Technical capability for the implementation of on-the-fly calibration through the possibility to push data directly from each sensor node to the “R” statistical package, where calibration curves and other post-processing can be done.

We will focus our future work on implementing the use case scenarios, as described in Section 4. Particular emphasis would be put on learning and documenting the experiences from the implementation of the use cases, which might lead to an improvement of the individual components and their interdependencies.

In terms of hardware and software, AirSensEUR has been developed to prioritize modularity and fast development time, thus trading power efficiency and component costs with design change requirements. By focusing on aspects of the system that have been already consolidated, a set of improvements can be implemented for the hardware and software infrastructure. For example, on the software side, especially for applications running on the host, translating parts of the currently-existing Java-based code to plain C or C++ will significantly reduce the computational costs on the main CPU and, consequently, the overall power consumption. The AirSensEUR hardware can benefit from component improvements, like, for example, in the A/D conversion area, thus reducing the number of onboard generated voltage references and power supply, or by introducing powerful microcontrollers, thus increasing the complexity of onboard filtering algorithms for better analysis performances. New sensors will be connected through the available communication peripherals or via modifications of the existing data protocol that would allow for several shields to be chained together. Modularity can also be improved via low cost specialized shields able to accommodate a single sensor.

The open nature of AirSensEUR will benefit from new communication technologies and standards, especially targeted to IoT, which could reduce the total system power consumption and operational costs and increase data accessibility. Last, but not least, the release of the code in the open source community and the sharing of experiences in the use of the platform will harness the creativity of the community and lead to collective improvements.

## Figures and Tables

**Table 1:** Hardware components of AirSensEUR.

1. Host with CPU at the back side	2. Sensor shield
3. Control panel	4. Battery
5. GPS Antenna	6. USB for GSM/GPS key
7. USB WiFi key	8. On/Off switch
9. USB power supply and battery recharging	9a. Wall power supply (220 V)
10. USB to Linux console to control CPU	

**Table 2:** Open source software products used in AirSensEUR.

Functionality	Products	Overview
1. Web transactions	AirSensEUR SOS-T client	Java application, pushing data (JSON POST transactions) from the host to a server when an Internet connection is available.
2. Storage	sqlite3	Local data storage on the sensor host.
	PostgreSQL/PostGIS	Server-side storage, with a database schema suitable for the $52{\;}^{\circ }\text{North}$ SOS implementation

3. Web services	$52{\;}^{\circ }\text{North}$ SOS	Implementation of an INSPIRE-compliant SOS
	$52{\;}^{\circ }\text{North}$ TimeSeriesAPI	RESTful interface on top of the SOS web service
4. Clients	$52{\;}^{\circ }\text{North}$ SensorWeb client	Mobile-friendly web client for interaction with observation data
	Geoserver	Mash-up with other geospatial data and implementation of INSPIRE discovery and view services
	RStudio (including Shiny and sensorweby)	JavaScript SOS client with functionality to process and analyze air quality data with R [29]
5. Visualization	R	Post-processing of data (e.g., for calibration or further statistical analysis)
